# Incidence of breast cancer subtypes in immigrant and non-immigrant women in Norway

**DOI:** 10.1186/s13058-021-01498-5

**Published:** 2022-01-10

**Authors:** Kirsti V. Hjerkind, Anna L. V. Johansson, Cassia B. Trewin, Hege G. Russnes, Giske Ursin

**Affiliations:** 1 Department of Registration, Cancer Registry of Norway, Oslo, Norway; 2 Department of Medical Epidemiology and Biostatistics, Karolinska Institute, 171 77 Stockholm, Sweden; 3 Cancer Registry of Norway, Postbox 5313, 0304 Majorstuen, Oslo, Norway; 4 Department of Cancer Genetics, Institute for Cancer Research, Oslo University Hospital, 0424 Oslo, Norway; 5 Department of Pathology, Oslo University Hospital, 0424 Oslo, Norway; 6 Department of Preventive Medicine, Keck School of Medicine, University of Southern California, Los Angeles, CA USA; 7 Department of Nutrition, Institute of Basic Medical Sciences, University of Oslo, Oslo, Norway

**Keywords:** Breast cancer subtypes, Immigrants, Incidence

## Abstract

**Background:**

Breast cancer incidence differs between non-immigrants and immigrants from low- and middle-income countries. This study investigates whether immigrants also have different subtype-specific incidences.

**Methods:**

We used national health registries in Norway and calculated subtype-specific incidence rate ratios (IRRs) for invasive breast cancer among women aged 20–75 and 20–49 years between 2005 and 2015. Immigrant groups were classified by country of birth broadly defined based on WHO regional groupings. Subtype was defined using estrogen receptor (ER), progesterone receptor (PR) and human epidermal growth factor 2 (HER2) status as luminal A-like (ER+ PR+ HER2-), luminal B-like/HER2- (ER+ PR- HER2-), luminal B-like/HER2+ (ER+ PR any HER2+), HER2+ (ER-PR-HER2+) and triple-negative breast cancer (TNBC) (ER-PR-HER2-).

**Results:**

Compared to non-immigrants, incidence of the luminal A-like subtype was lower in immigrants from Sub-Saharan Africa (IRR 0.43 95% CI 0.28–0.66), South East Asia (IRR 0.63 95% CI 0.51–0.79), South Asia (IRR 0.67 95% CI 0.52–0.86) and Eastern Europe (IRR 0.86 95% CI 0.76–0.99). Immigrants from South Asia had higher rates of HER2 + tumors (IRR 2.02 95% CI 1.26–3.23). The rates of TNBC tended to be similar regardless of region of birth, except that women from South East Asia had an IRR of 0.54 (95% CI 0.32–0.91).

**Conclusions:**

Women from Eastern Europe, Sub-Saharan Africa and Asia had different subtype-specific incidences compared to women from high-income countries (including non-immigrants). These differences in tumor characteristics between immigrant groups should be taken into consideration when planning preventive or screening strategies.

**Supplementary Information:**

The online version contains supplementary material available at 10.1186/s13058-021-01498-5.

## Background

Breast cancer incidence varies across the world with age-standardized incidence rates (ASRs) above 73 cases per 100,000 person-years in Western Europe, the United States (U.S.), Canada, Australia and New Zealand and below 34 cases per 100,000 person-years in most of Sub-Saharan Africa, South Asia and South-East Asia [1]. Breast cancer can be subdivided into many different subtypes. Historically, these were defined using results on hormone receptor status (estrogen receptor [ER] and progesterone receptor [PR]) from immunohistochemical (IHC) analyses, and subsequently human epidermal growth factor 2 (HER2) status was added. Although these subtypes do not completely overlap the subtypes based on molecular expression studies [2], clinical practice still depends largely on these biomarkers. The different breast cancer subtypes represent distinct biological and clinical behaviors; some have more aggressive behavior and worse prognosis, and they respond differently to treatment options [3].

Studies from Africa indicate that African women have a high proportion of high grade tumors with an aggressive subtype, e.g., triple-negative breast cancer (TNBC) [4, 5]. These tumors develop at a young age with an advanced stage distribution [5].

Many studies from the U.S. have demonstrated differences in incidence and survival across racial/ethnic groups (African-American, Hispanic and South-Asian women compared to non-Hispanic White women) [3, 6–11]. These racial/ethnic groups are not directly comparable to the immigrant groups most commonly seen in Europe. However, an interesting question is whether there are similar differences in incidence and survival between European immigrant groups. We have previously reported that the incidence of stage-specific breast cancer was lower in immigrants than in non-immigrants in Norway [12]. Another Norwegian study found that immigrant women from low- and middle-income countries may have more advanced stage of breast cancer than non-immigrant women [13]. There are not many studies investigating subtype distribution among immigrant women with breast cancer in Western Europe. One smaller study found that Arab immigrants in Europe tend to develop cancer at a younger age with more luminal B-like and less luminal A-like subtypes than European women [14].

Socioeconomic status (SES) contributes to the racial/ethnic disparities in breast cancer incidence and survival in the U.S. [15, 16]. Such differences across SES could be due to health care access and could also be present across immigrant groups in Europe. On the other hand, there may be less of a difference when access to health care is universal, or where there are organized screening programs.

We previously reported that breast cancer incidence in Norway is higher in non-immigrant women and immigrant women from Western Europe and North America, and lower in women from Asia and Sub-Saharan Africa [17]. This study investigates breast cancer subtypes among immigrant groups using national health data in Norway from 2005 to 2015.

## Methods

### Cohort data

We used national Norwegian population registries to define a cohort of women which was linked with the Cancer Registry of Norway. Eligible women were registered as residents of Norway for twelve or more months during the period 2005–2015 (*n* = 3,329,630). We linked registries using the personal identification number (PIN) assigned to Norwegian-born at birth and to immigrants within six months after immigration. Information on vital status, including date of death and date of emigration, country of birth, date of immigration, and SES, such as education, household income and number of people in the household, was obtained from the Norwegian Population Registry at Statistics Norway.

### Definition of immigrant groups

Immigrants were defined as individuals born outside of Norway with two foreign-born parents and a registered date of immigration (first generation immigrants), and non-immigrants were defined as individuals born in Norway or abroad with one or two Norwegian-born parents. Included in the non-immigrant population were individuals born in Norway of immigrant parents (second generation immigrants) (0.8% of the study population). The immigrants were classified according to their country of birth, which were collapsed into regions broadly defined consistent with the WHO regional groupings (Non-immigrants; Immigrants from high-income countries including Western Europe, U.S.A., Canada, Australia, and New Zealand; Eastern Europe including Eastern Europe, Baltics and Balkan countries; Middle East including the Middle East and North Africa; Sub-Saharan Africa; South Asia; South-East Asia, Additional file 1: Table S1).

### Ascertainment of breast cancer diagnoses

We identified breast cancer cases in the cohort during follow-up (January 1 2005 to December 31 2015) by linking the cohort to the Cancer Registry of Norway. A primary first invasive breast cancer was classified according to the 10th revision of the International Classification of Diseases (ICD-10), code C50. The Cancer Registry of Norway has since 1952 systematically collected notifications on cancer occurrence for the Norwegian population, reporting has been mandatory by law since the start, and the registry is considered to be close to complete [18].

From the 3,329,630 eligible women, we excluded 9741 women who had a previous diagnosis of breast cancer and 43,457 women from countries outside the included birth regions (South America and Eastern Asia due to few cases of breast cancer [*n* = 181]). There were very few immigrants over 75 years in Norway and no subtype information available in this age group because of internal coding priorities at the Cancer Registry of Norway. Also, breast cancer is not common in individuals below the age of 20 years. Hence, we excluded women outside the age span 20–75 years leaving 1,921,487 women aged 20–75 years for the analysis. Since we did not have information on mode of detection in the current dataset, we also created a non-screened population by restricting to women aged 20–49 years who are not yet in screening age (1,349,942 women).

### Ascertainment of breast cancer subtypes

Since 2005, the Cancer Registry of Norway collects information on hormone receptor status (ER, PR) and HER2 based on IHC results from pathology reports for women with breast cancer. From 2005 to January 2012, tumors were classified as ER- if there was <10% reactivity. From February 2012, the threshold for ER- tumors was changed to <1% reactivity as a result of change in the treatment protocols for patients attending clinics in Norway. PR- tumors were defined as those with reactivity of < 10%, and PR+ tumors as those with reactivity ≥10% throughout the study period. HER2 expression status was routinely assessed with IHC and in general with in situ hybridization if the IHC results were borderline. Breast cancer subtypes were defined by IHC surrogates for molecular subtype according to the St Gallen 2013 criteria without using Ki67: luminal A-like (ER+ PR+ HER2-) *n* = 12,568, luminal B-like/HER2- (ER+ PR- HER2-) *n* = 2984, luminal B-like/HER2+ (ER+ PR any HER2+) *n* = 2205, HER2+ (ER- PR- HER2+) *n* = 1068 and TNBC (ER- PR- HER2-) *n* = 2050 [19, 20]. Subtype was set to “unknown” if any of ER, PR or HER2 were missing, or if ER- PR+ HER2 any.

### Statistical methods

We used Poisson regression to estimate incidence rate ratios of invasive breast cancer by subtype and across regions of birth. Individuals were followed from age 20 or date of immigration, whatever occurred last, until diagnosis of first invasive breast cancer, death, emigration, age 76 or December 31, 2015, whichever occurred first. In the analysis of ages 20–49 the time-to-event was censored at age 50. The outcome was time to first breast cancer, hence in the estimated incidence of e.g., luminal A-like breast cancer, only women with a first breast cancer which was luminal A-like contributed events, while women with a first breast cancer which was another subtype or unknown were censored at first breast cancer. Time-to-event of the other subtypes was defined similarly. We estimated age-standardized incidence rates (ASRs) of subtype-specific breast cancer across regions of birth using the age distribution from the world standard population [21]. To compare the immigrant groups to non-immigrants, we estimated subtype-specific incidence rate ratios (IRRs) and confidence intervals (CIs) using Poisson regression models with regions of birth as the main covariate and with adjustment for age during follow-up in 5-year categories (20-24 years, 25-29 years, etc.) after time-splitting. The IRRs conveyed the relative risk of developing a particular subtype for an immigrant group compared with the non-immigrant population, and were estimated for ages 20–75 and 20–49 years separately. In a second step, we also performed separate age-adjusted Poisson regression models by grade for all subtypes combined and within luminal A-like breast cancer (Table 3), and by ER status (Table 4).

### Additional analyses

In sensitivity analyses, we adjusted the Poisson models for the most recently recorded education level before diagnosis, and categorized as compulsory (≤ 10 years), secondary (11–13 years) or tertiary (≥ 14 years) education. We also adjusted for average household income in quintiles, collected during the 5-year period prior to cancer diagnosis. We did not include either of these covariates in the final analyses since they did not change the results notably. To assess the impact of unknown subtype, we performed a sensitivity analysis restricting to the diagnosis period 2010–2015, where proportion of unknown subtype was low (6.8%) among both immigrant and non-immigrant groups. The sensitivity analysis included all subtypes combined and the luminal A-like subtype. Finally, we restricted the analysis to women aged 20–40 years for all subtypes combined and for the luminal A-like subtype.

All statistical analyses were carried out using Stata version 15.1 (StataCorp. 2017. *Stata Statistical Software: Release 15*. College Station, TX: StataCorp LLC). The study was approved by the Regional Committees for Medical and Health Research Ethics (no. 2013/2376-17).

## Results

The study population included 1,611,371 non-immigrant women (88.7% of the person-years) and 310,116 immigrant women (11.3% of the person-years) aged 20–75 years. Table 1 shows descriptive characteristics by immigrant subgroups. Mean age at immigration was similar across regions of birth, while the year of immigration, education level and income differed. Age at onset of breast cancer differed across regions of birth, likely reflecting differences in follow-up available due to year of immigration.

**Table 1:** Characteristics of the study population by region of birth in women aged 20–75 years

	Non-immigrants	Immigrants					
	Non-immigrants	High-income	Eastern Europe	Middle East	Sub-Saharan Africa	South Asia	South East Asia
*Total population*							
Mean age (SD) at immigration	N/A	26.2 (12.4)	30.0 (13.3)	24.2 (14.1)	23.3 (12.4)	25.0 (14.2)	27.4 (10.9)
Mean year (SD) of immigration	N/A	1995 (17)	2005 (10)	2003 (9)	2006 (8)	2000 (12)	2004 (10)
Education (% tertiary)*	14.1	47.5	45.9	34.2	20.7	32.5	59.5
Income (% lowest quintile)*	14.0	31.7	41.6	62.2	75.4	44.3	39.3
*Cases only*							
Mean age (SD) when diagnosed	62.2 (14.2)	60.9 (13.6)	51.8 (12.3)	49.1 (11.8)	44.8 (11.2)	51.9 (12.3)	48.1 (10.2)
Mean time (SD) from immigration to diagnosis, years	N/A	30.4 (14.9)	14.8 (11.9)	13.3 (8.8)	14.5 (12.4)	19.9 (10.7)	16.1 (10.6)
Tumor grade (% high grade 3 or 4)	16.0	16.2	19.3	21.6	19.1	21.1	21.1

High-income; Western Europe, USA, Canada, Australia and New Zealand, Eastern Europe; Eastern Europe, Baltics and Balkan, Middle East; Middle East and North Africa, SD; standard deviation, diag; diagnosis

*Highest level of education or income achieved during the study period

### Distribution of breast cancer subtypes

**Fig. 1:**
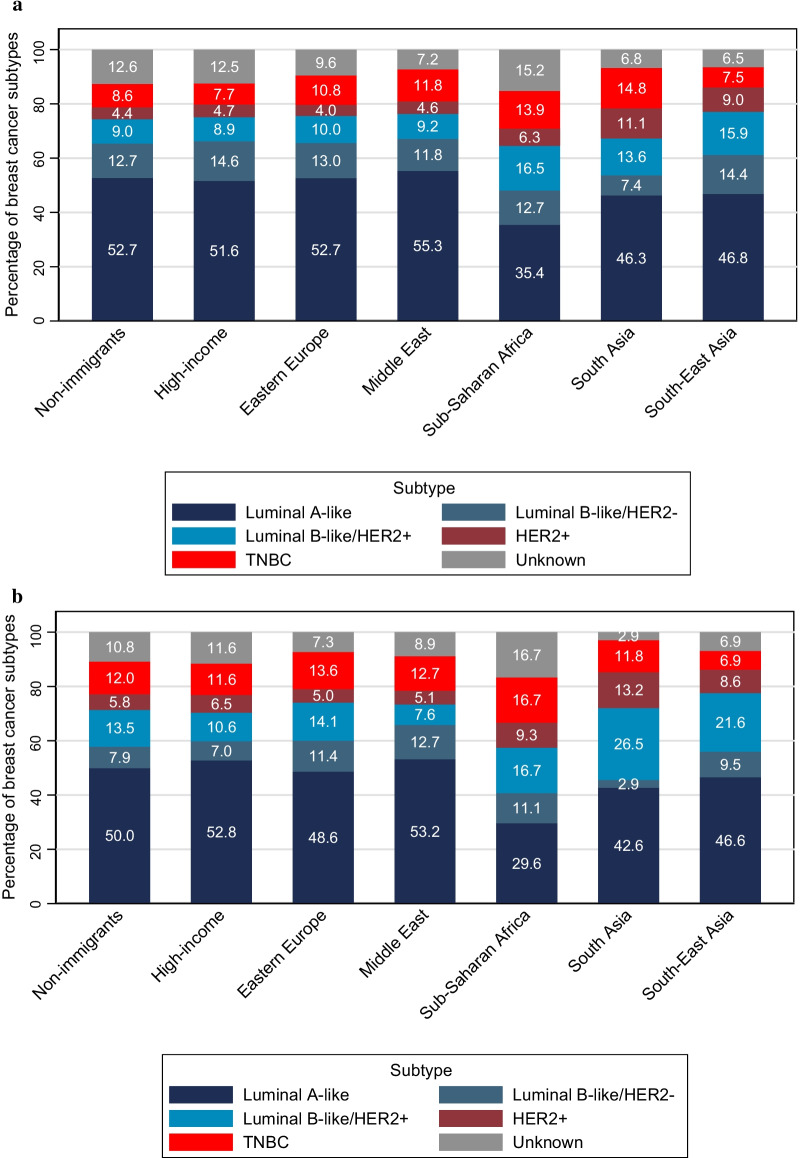
**a** Distribution of subtypes within regions of birth in women aged 20–75 years. **b** Distribution of subtypes within regions of birth in women aged 20–49 years. High-income; Western Europe, USA, Canada, Australia and New Zealand, Eastern Europe; Eastern Europe, Baltics and Balkan, Middle East; Middle East and North Africa, TNBC; triple-negative breast cancer

The luminal A-like subtype was the most common both in non-immigrants and immigrant groups (Fig. 1a). The percentage of the luminal A-like subtype was especially high in non-immigrants and women from high-income countries, as well as women from Eastern Europe and the Middle East. The luminal B-like HER2+ subtype was more common in Sub-Saharan and in Asian women, and the HER2+ subtype more common in Asian women. The TNBC subtype was less common in non-immigrant women and women from high-income countries and South-East Asian countries. These distributions were similar in the subgroup of women aged 20–49 years, although women from Asia had even more of the luminal B-like HER2+ subtype, and Sub-Saharan women more and Asian women less of the TNBC subtype (Fig. 1b). The percentage of unknown subtype was lowest in South Asian and South-East Asian women.

### Age-standardized rates within subtypes

**Fig. 2:**
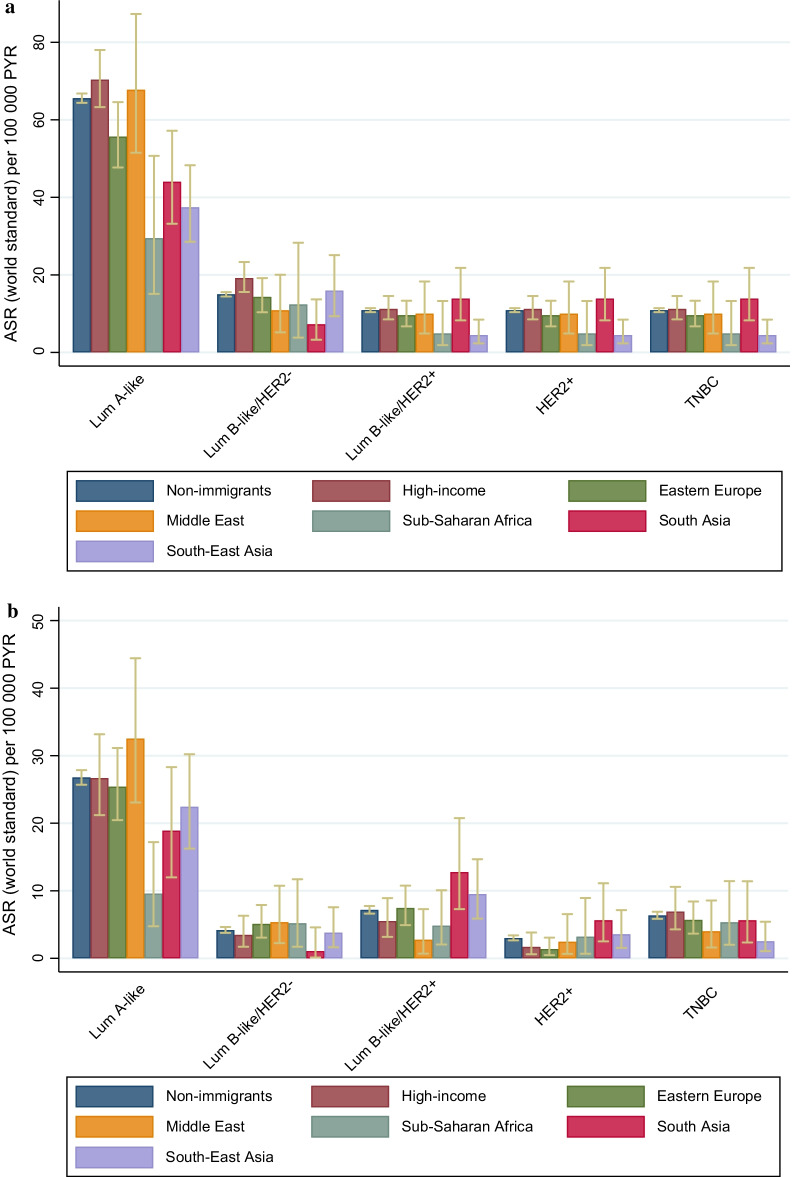
**a** Age-standardized (world standard) incidence rates for different subtypes by region of birth in women aged 20–75 years. **b** Age-standardized (world standard) incidence rates for different subtypes by region of birth in women aged 20–49 years. ASR; age-standardized rate, PYR; person years, High-income; Western Europe, USA, Canada, Australia and New Zealand, Eastern Europe; Eastern Europe, Baltics and Balkan, Middle East; Middle East and North Africa, Lum A; luminal A-like, Lum B; luminal B-like, TNBC; triple-negative breast cancer

For the luminal-A like subtype, women from high-income countries had the highest ASR and Sub-Saharan women the lowest (Fig. 2a). Women from South Asia had the highest ASR of the HER2+ and TNBC subtypes, while women from Sub-Saharan Africa and South-East Asia had the lowest ASR. In ages 20–49 years, women from Sub-Saharan Africa had the lowest ASR of luminal A-like subtype, while women from South Asia and South-East Asia had the highest ASR of the luminal B-like HER2 + and HER2 + subtypes (Fig. 2b).

### Age-adjusted incidence rate ratios

In models adjusted for age, women from high-income countries (IRR 1.07 95% CI 0.99–1.16) and from the Middle-East (IRR 0.93 95% CI 0.79–1.10) had similar rates of breast cancer compared to non-immigrants (Table 2). Women from Eastern Europe, Sub-Saharan Africa and Asia had significantly decreased rates of cancer compared to non-immigrants. This finding was similar in women aged 20–75 and women aged 20–49, however not significant in the latter. For the luminal A-like subtype, women from Sub-Saharan Africa had a lower rate compared to non-immigrant women, with an IRR of 0.43 (95% CI 0.28–0.66) in the age group 20–75 years and an IRR of 0.44 (95% CI 0.25–0.78) in the age group 20–49 years. Within the luminal B-like HER2- subtype, women from high-income countries aged 20–75 had a higher rate (IRR 1.29 95% CI 1.06–1.57) compared to non-immigrant women, whereas women from the other regions had about the same rate, except women from South Asia who had a lower rate (IRR 0.49 95% CI 0.27–0.92). The pattern was similar among women aged 20–49, but the differences were not statistically significant in this group. Within the luminal B-like HER2+ subtype, immigrant women from all regions aged 20–75 years had almost the same rates as non-immigrant women. Similar results were found in women aged 20–49, with the exception of South Asian women who had a significantly higher rate (IRR 1.72 95% CI 1.06–2.78). Within the HER2+ subtype, Eastern European women aged 20–75 years had a significantly lower rate of the HER2+ subtype compared to non-immigrant women of the same age (IRR 0.51 0.30–0.89). South Asian women aged 20–75 years had a significantly higher rate (IRR 2.02 95% CI 1.26–3.23), as did South Asian women aged 20–49 years (IRR 2.13 95% CI 1.09–4.13). Within the TNBC subtype, immigrant women from the different regions had almost the same rate as non-immigrant women. The exception was women from South-East Asia, who had half the rate compared to non-immigrant women (20–75 years: IRR 0.54 [95% CI 0.32–0.91] and 20–49 years: IRR 0.50 [95% CI 0.25–1.01]).

**Table 2:** Incidence Rate Ratios (IRRs) for country of birth and invasive breast cancer subtypes

	Age 20–75		Age 20–49	
	Cases/PYR	IRR (95% CI)	Cases/PYR	IRR (95% CI)
*All subtypes*				
Non-immigrants	21,682/14608517	1.00 (ref)	4750/8335077	1.00 (ref)
High-income	675/477618	1.07 (0.99–1.16)	154/305153	0.94 (0.80–1.10)
Eastern Europe	417/593550	0.85 (0.77–0.93)	195/486627	0.90 (0.78–1.04)
Middle East	136/176786	0.93 (0.79–1.10)	69/147450	0.97 (0.77–1.23)
Sub-Saharan Africa	60/152194	0.62 (0.48–0.80)	43/136880	0.75 (0.56–1.02)
South Asia	139/180867	0.78 (0.66–0.92)	61/139461	0.87 (0.68–1.12)
South-East Asia	178/284409	0.71 (0.61–0.82)	101/232898	0.85 (0.70–1.04)
*Luminal A-like*				
Non-immigrants	11,732/14608517	1.00 (ref)	2418/8335077	1.00 (ref)
High-income	366/477618	1.08 (0.97–1.20)	84/305153	1.03 (0.83–1.28)
Eastern Europe	222/593550	0.86 (0.76–0.99)	99/486627	0.95 (0.77–1.16)
Middle East	81/176786	1.06 (0.85–1.32)	41/147450	1.18 (0.86–1.60)
Sub-Saharan Africa	21/152194	0.43 (0.28–0.66)	12/136880	0.44 (0.25–0.78)
South Asia	63/180867	0.67 (0.52–0.86)	25/139461	0.73 (0.49–1.08)
South-East Asia	83/284409	0.63 (0.51–0.79)	46/232898	0.79 (0.59–1.06)
*Luminal B-like/HER2-*				
Non-immigrants	2767/14608517	1.00 (ref)	377/8335077	1.00 (ref)
High-income	102/477618	1.29 (1.06–1.57)	11/305153	0.85 (0.46–1.54)
Eastern Europe	55/593550	1.02 (0.78–1.33)	21/486627	1.20 (0.77–1.87)
Middle East	15/176786	0.97 (0.58–1.61)	8/147450	1.41 (0.70–2.83)
Sub-Saharan Africa	9/152194	0.93 (0.48–1.80)	6/136880	1.30 (0.58–2.92)
South Asia	10/180867	0.49 (0.27–0.92)	2/139461	0.36 (0.09–1.43)
South-East Asia	26/284409	0.97 (0.66–1.44)	9/232898	0.95 (0.49–1.84)
*Luminal B-like/HER2*+				
Non-immigrants	2030/14608517	1.00 (ref)	653/8335077	1.00 (ref)
High-income	58/477618	0.96 (0.74–1.25)	17/305153	0.75 (0.46-.21)
Eastern Europe	46/593550	0.84 (0.63–1.13)	30/486627	0.97 (0.67–1.40)
Middle East	12/176786	0.72 (0.41–1.28)	4/147450	0.40 (0.15–1.06)
Sub-Saharan Africa	10/152194	0.85 (0.46–1.59)	8/136880	0.97 (0.48–1.94)
South Asia	20/180867	1.05 (0.68–1.63)	17/139461	1.72 (1.06–2.78)
South-East Asia	29/284409	1.03 (0.71–1.49)	23/232898	1.37 (0.90–2.08)
*HER2*+				
Non-immigrants	984/14608517	1.00 (ref)	274/8335077	1.00 (ref)
High-income	27/477618	0.93 (0.63–1.36)	6/305153	0.62 (0.28–1.40)
Eastern Europe	13/593550	0.51 (0.30–0.89)	6/486627	0.45 (0.20–1.01)
Middle East	6/176786	0.79 (0.35–1.76)	4/147450	0.93 (0.35–2.49)
Sub-Saharan Africa	4/152194	0.75 (0.28–2.01)	4/136880	1.11 (0.41–2.99)
South Asia	18/180867	2.02 (1.26–3.23)	9/139461	2.13 (1.09–4.13)
South-East Asia	16/284409	1.22 (0.75–2.01)	9/232898	1.24 (0.64–2.42)
*TNBC*				
Non-immigrants	1886/14608517	1.00 (ref)	582/8335077	1.00 (ref)
High-income	59/477618	1.03 (0.79–1.34)	23/305153	1.10 (0.73–1.67)
Eastern Europe	47/593550	0.93 (0.69–1.24)	27/486627	0.92 (0.62–1.35)
Middle East	14/176786	0.92 (0.54–1.56)	7/147450	0.74 (0.35–1.56)
Sub-Saharan Africa	9/152194	0.82 (0.43–1.58)	8/136880	1.00 (0.50–2.01)
South Asia	21/180867	1.19 (0.78–1.84)	8/139461	0.87 (0.43–1.79)
South-East Asia	14/284409	0.54 (0.32–0.91)	8/232898	0.50 (0.25–1.01)
*Unknown*				
Non-immigrants	2469/14608517	1.00 (ref)	509/8335077	1.00 (ref)
High-income	69/476027	0.97 (0.76–1.23)	15/305153	0.86 (0.51–1.43)
Eastern Europe	38/593550	0.68 (0.50–0.94)	13/486627	0.55 (0.32–0.96)
Middle East	9/176786	0.55 (0.28–1.06)	5/147450	0.65 (0.27–1.58)
Sub-Saharan Africa	8/152194	0.75 (0.37–1.49)	6/136880	0.97 (0.43–2.18)
South Asia	8/180867	0.40 (0.20–0.79)	1/139461	0.13 (0.02–0.94)
South-East Asia	12/284409	0.43 (0.24–0.75)	7/232898	0.55 (0.26–1.15)

PYR; person-years, IRR; incidence rate ratio, CI; confidence interval, ref; reference, High-income; Western Europe, USA, Canada, Australia and New Zealand, Eastern Europe; Eastern Europe, Baltics and Balkan, Middle East; Middle East and North Africa

### Age-adjusted incidence rate ratios by grade

For all subtypes, women aged 20–75 years from Sub-Saharan Africa, South Asia and South-East Asia had decreased rates of grade I and grade II tumors relative to the non-immigrant reference group (Table 3). Eastern European women also had lower rates of grade I tumors (20–75 years: IRR 0.70 (95% CI 0.54–0.90). For grade III tumors, the rates across all immigrant groups were almost the same as in non-immigrants. Similar trends were observed for the luminal A-like subtype and among women aged 20–49 years.

**Table 3:** Incidence Rate Ratios (IRRs) for country of birth and invasive breast cancer by grade

	Age 20–75						Age 20–49					
	Grade I		Grade II		Grade III		Grade I		Grade II		Grade III	
	Cases	IRR (CI)	Cases	IRR (CI)	Cases	IRR (CI)	Cases	IRR (CI)	Cases	IRR (CI)	Cases	IRR (CI)
*All subtypes*												
Non-immigrants	4496	1.00 (ref)	9793	1.00 (ref)	5743	1.00 (ref)	605	1.00 (ref)	1981	1.00 (ref)	1776	1.00 (ref)
High-income	126	0.99 (0.83–1.18)	313	1.10 (0.99–1.24)	177	1.04 (0.90–1.21)	20	0.99 (0.63–1.55)	61	0.89 (0.69–1.16)	63	1.01 (0.79–1.30)
Eastern Europe	61	0.70 (0.54–0.90)	189	0.89 (0.77–1.02)	133	0.87 (0.74–1.04)	16	0.63 (0.38–1.04)	84	0.96 (0.77–1.20)	79	0.92 (0.74–1.16)
Middle East	21	0.83 (0.54–1.28)	54	0.85 (0.65–1.12)	50	1.09 (0.82–1.44)	8	0.94 (0.47–1.89)	29	1.00 (0.70–1.45)	27	0.97 (0.67–1.42)
Sub-Saharan Africa	2	0.13 (0.03–0.53)	19	0.47 (0.30–0.73)	32	0.98 (0.69–1.39)	1	0.15 (0.02–1.10)	12	0.53 (0.30–0.93)	27	1.17 (0.80–1.72)
South Asia	8	0.24 (0.12–0.48)	56	0.72 (0.55–0.93)	61	1.15 (0.89–1.48)	1	0.12 (0.02–0.85)	21	0.74 (0.48–1.13)	30	1.10 (0.77–1.58)
South-East Asia	27	0.62 (0.42–0.90)	64	0.59 (0.46–0.76)	74	0.94 (0.75–1.19)	10	0.71 (0.38–1.32)	44	0.91 (0.68–1.23)	41	0.88 (0.65–1.20)
*Luminal A-like*												
Non-immigrants	3311	1.00 (ref)	6154	1.00 (ref)	1645	1.00 (ref)	473	1.00 (ref)	1316	1.00 (ref)	486	1.00 (ref)
High-income	99	1.05 (0.86–1.29)	196	1.10 (0.95–1.27)	47	0.99 (0.74–1.32)	19	1.20 (0.76–1.90)	42	0.95 (0.70–1.29)	17	1.02 (0.63–1.65)
Eastern Europe	48	0.73 (0.55–0.97)	124	0.92 (0.77–1.10)	37	0.88 (0.64–1.22)	14	0.71 (0.42–1.20)	54	0.96 (0.73–1.26)	24	1.08 (0.71–1.62)
Middle East	17	0.89 (0.55–1.44)	41	1.01 (0.75–1.38)	17	1.33 (0.82–2.14)	6	0.90 (0.40–2.02)	22	1.17 (0.77–1.78)	11	1.50 (0.82–2.72)
Sub-Saharan Africa	2	0.17 (0.04–0.69)	7	0.27 (0.13–0.57)	10	1.13 (0.60–2.10)	1	0.20 (0.03–1.41)	3	0.21 (0.07–0.64)	8	1.35 (0.67–2.71)
South Asia	8	0.32 (0.16–0.65)	38	0.76 (0.56–1.05)	15	1.01 (0.61–1.68)	1	0.15 (0.02–1.08)	13	0.70 (0.40–1.21)	9	1.25 (0.65–2.41)
South-East Asia	21	0.64 (0.42–0.98)	45	0.65 (0.49–0.87)	15	0.69 (0.41–1.15)	7	0.63 (0.30–1.34)	30	0.96 (0.67–1.38)	9	0.74 (0.38–1.43)

IRR; incidence rate ratio, CI; confidence interval, ref; reference, High-income; Western Europe, USA, Canada, Australia and New Zealand, Eastern Europe; Eastern Europe, Baltics and Balkan, Middle East; Middle East and North Africa

### Age-adjusted incidence rate ratios by ER status

To provide more robust estimates, we also examined whether region of birth had an effect on a cruder subtype division, simply ER status (Table 4). Sub-Saharan women had lower rates of ER+ tumors compared to the non-immigrant reference groups, both within ages 20–75 years (IRR 0.57 95% CI 0.42–0.77) and 20–49 years (IRR 0.66 95% CI 0.46–0.96). South Asian (IRR 0.67 95% CI 0.42–0.77) and South-East Asian (IRR 0.71 95% CI 0.60–0.84) women aged 20–75 years also had lower rates of ER+ tumors compared to the non-immigrant reference group, and South Asian women had a higher rate of ER- tumors, however only borderline significant (IRR 1.36 95% CI 1.00–1.85). Women from Eastern Europe aged 20–75 years had lower rates of both ER- (IRR 0.76 95% CI 0.60–0.98) and ER+ (IRR 0.86 95% CI 0.77–0.95) tumors, compared to the non-immigrant reference group.

**Table 4:** Incidence Rate Ratios (IRRs) for country of birth and invasive breast cancer by ER status

	Age 20–75				Age 20–49			
	ER-		ER +		ER-		ER +	
	Cases	IRR (CI)	Cases	IRR (CI)	Cases	IRR (CI)	Cases	IRR (CI)
Non-immigrants	3282	1.00 (ref)	17,844	1.00 (ref)	959	1.00 (ref)	3645	1.00 (ref)
High-income	94	0.95 (0.78–1.17)	561	1.09 (1.00–1.18)	29	0.85 (0.59–1.23)	120	0.97 (0.81–1.16)
Eastern Europe	66	0.76 (0.60–0.98)	336	0.86 (0.77–0.95)	35	0.73 (0.52–1.03)	155	0.96 (0.82–1.12)
Middle East	23	0.89 (0.59–1.34)	110	0.95 (0.78–1.14)	13	0.85 (0.49–1.47)	55	1.03 (0.79–1.34)
Sub-Saharan Africa	13	0.71 (0.41–1.22)	43	0.57 (0.42–0.77)	12	0.93 (0.53–1.65)	28	0.66 (0.46–0.96)
South Asia	41	1.36 (1.00–1.85)	96	0.67 (0.55–0.82)	16	1.06 (0.65–1.75)	45	0.85 (0.64–1.14)
South-East Asia	31	0.70 (0.49–1.00)	142	0.71 (0.60–0.84)	18	0.70 (0.44–1.11)	79	0.89 (0.71–1.11)

ER; estrogen receptor, IRR; incidence rate ratio, CI; confidence interval, ref; reference, High-income; Western Europe, USA, Canada, Australia and New Zealand, Eastern Europe; Eastern Europe, Baltics and Balkan, Middle East; Middle East and North Africa

### Sensitivity analyses by unknown subtype and young age

The proportion of unknown subtype depended on year of diagnosis and was lower in the later periods. More Asian women were diagnosed from 2010 onwards. Subtype was not available during 2005–2009 for the age group 70–75 years, which had few immigrants, so non-immigrant women had the highest proportion of unknown subtype in our data (12.5%). In Additional file 1: Table S2 we assessed the impact of unknown subtype by restricting to the diagnosis period 2010–2015 and found estimates to be very similar to results from the full study period 2005–2015.

To minimize the possible influence of mammographic screening on our results, we assessed the effect of country of birth in a subgroup of women aged 20–40 years. The Norwegian Breast Cancer Screening Program targets women from age 50 years, and mammograms before age 45 in the Norwegian population are rare. This subgroup analysis yielded similar results as for 20–75 years (Additional file 1: Table S3).

## Discussion

We found that, in Norway, immigrant women from high-income countries had the highest incidence of breast cancer overall and of similar level as non-immigrant women. Luminal A-like breast cancer was the most common subtype regardless of region of birth. Compared to non-immigrant women, women from Eastern Europe, Sub-Saharan Africa and Asia had lower rates of the luminal A-like subtype, while Asian women had higher rates of the HER2+ subtype. The rates of TNBC tended to be similar regardless of region of birth, except in women from South-East Asia who had half the rate. Women from Sub-Saharan Africa and Asia were less likely to have grade I and grade II tumors and ER+ tumors, and women from South Asia more likely to have ER- tumors, compared to non-immigrant women. These differences were primarily driven by incidence above the age of 50 years, however, results were similar in the subgroup of women aged 20–49 years.

In line with our findings, others have found that women from Western Europe are prone to the more common luminal subtypes [22], while women from Asia and Africa have lower incidence of especially the luminal A-like subtype [5, 23]. In particular, TNBC appears to be common in African women [4, 5], while HER2+ cancers may be more common in Asians. One study from New Zealand found Asian women to be more likely to have grade III and HER2+ breast cancers compared to women of European ancestry [24]. Another study found Arab women living in Australia to be younger and have more tumors of high grade and HER2+ subtype compared to non-immigrant women [25].

In Asian countries, incidence rates have been increasing from a young age in later cohorts, which for a while led to different age-incidence curves in Asia than in other parts of the world. This birth-cohort effect has been well described [26, 27] and the increase is likely partly due to changes in lifestyle over time, and partly to increased screening. Regardless, our study indicates that subtypes among the Asian immigrants in Norway are still somewhat dissimilar to non-immigrants, with more HER2+ breast cancer among Asian immigrants.

Some of the observed differences across immigrant groups described above are similar to those described across broad racial/ethnic groups (African-American, Asian-American, Hispanic and non-Hispanic White) in the U.S. [3, 7–11]. In particular, luminal cancers are more common in Non-Hispanic Whites [3, 28], African-Americans have more TNBC [28] and Asian-Americans have higher rates of HER2+ cancers [29, 30].

The incidence of all breast cancer subtypes increase with age, but some subtypes have less of an incline with age than others [31]. The greatest increase in incidence with age is for the ER+ or luminal subtypes. This means that among young women, HER2+ and TNBC subtypes are proportionally more common [31–33]. Data from the U.S. suggest that the increase in incidence by age varies not only by subtype but *also* by racial/ethnic group [31, 34]. The increase in luminal subtypes by age tends to be most pronounced in non-Hispanic Whites, and could be due to a higher level of screening in non-Hispanic Whites in the U.S.

The observed differences in subtype-specific incidence across immigrant groups could, similarly to differences across racial/ethnic groups in the U.S., be due to a number of factors including variations in genetics, lifestyle, health seeking behavior or a combination. Data from the U.S. suggest that mutations in the breast cancer susceptibility genes BRCA1 and BRCA2 may be more prevalent in African-American compared to non-Hispanic White women [35], while mutations in other breast cancer genes may be less common in African-Americans [36]. There is evidence, although not completely consistent, that BRCA mutation carriers are more likely to develop TNBC [4, 37–39]. In our study we found similar absolute incidence of TNBC across all immigrant groups except for a lower incidence in women from Sub-Saharan Africa and South-East Asia. We do not know the prevalence of BRCA mutations in these breast cancer patients in Norway, however, one report found similar prevalence in Asians as in other ethnic groups [40]. Given the current state of knowledge it seems unclear how important genetic factors are for the subtype distributions we observed.

A high incidence of luminal cancers among non-immigrants and immigrants from high-income countries is probably due to a higher prevalence of classical breast cancer risk factors among these women, such as high age at first birth and low parity [20, 41], as well as increased alcohol consumption and obesity [42–44]. The effects of these risk factors are not consistently limited to luminal cancers, and some, including obesity, have also been associated with TNBC [6, 16, 45]. However, in general both TNBC and HER2+ cancers [46] seem less strongly associated with classical reproductive factors.

Discrepancies in access to health care could also result in subtype differences across immigrant groups. A previous study using Norwegian data from 1990 to 2014 found that immigrant women from low- and middle-income countries had more advanced stage of breast cancer than non-immigrant women. The results were, however, rather consistent in women above and under 50 years of age, suggesting that screening differences do not explain these results [13]. We also did not find any effect of SES on subtype-specific incidence. However, although Norway has organized screening and public health care, immigrants still tend to have differences in the use of health care [47]. Different screening practices across immigrant groups can therefore partially have explained our findings [48–50], since luminal A-like tumors are more frequent among women with screen-detected cancer [51]. On the other hand, we found results to be similar in the age group 20–49 years, an age group not offered breast cancer screening. Further, data from the Norwegian Breast Cancer Screening Program found that immigrant women from low- and middle-income countries with interval cancers were more likely to have a TNBC subtype compared to non-immigrants [52]. In addition, both among screen-detected and interval cancers, immigrants from low- and middle-income countries were more likely than non-immigrants to be diagnosed with grade 3 tumors. Thus, although we cannot exclude that part of our observed differences were due to differences in screening, we also cannot exclude the possibility that cancer may develop differently biologically in some immigrant groups.

After moving to a new country, immigrants go through different degrees of acculturation, and after one or two generations, they often adapt to the culture and lifestyle of their new home country [53]. Both lifestyle and screening participation may change with time and could contribute to more luminal cancers.

The strengths of this study include the large number of women from a geographically diverse population, maximum follow-up of 10 years, and the use of individual breast cancer subtypes. Norway has a predominantly white population with immigrants from many parts of the world. Residents have access to universal, publicly funded health care, including breast cancer screening, diagnosis and treatment.

A limitation of the study is the small number of events in some of the groups, and this produces at times uncertain effect estimates. Using more parsimonious groupings of both subtypes and countries of birth would provide more reliable results. However, as we and others have previously shown, there are substantial differences in both etiology and prognosis of cancers depending on these subtypes [20, 54, 55] and birth country groupings [17], and we therefore believe it is important to show more detailed subgroups.

The immigrant women are on average younger than the non-immigrant women, and this difference in age distribution between the immigrant and non-immigrant groups could have affected subtype distribution. However, the results were similar in the subgroup of women aged 20–49 years.

Time since immigration is likely important since immigration patterns to Norway have changed over time, depending mostly on the reasons for immigration (employment, family, study or seeking refuge). Immigrants in the 1970s and from 2004 and onwards were predominately workers from Pakistan, Yugoslavia and Turkey and from Poland and Lithuania, respectively. Immigrants in the 1990s and in 2015 were predominantly refugees from the Baltic countries and from Syria and Afghanistan, respectively [56, 57]. We could not adjust for time since immigration since we have relatively few cases and would need to categorize time since immigration in broad categories. Since different immigrant groups have arrived in Norway at different periods of time, country of birth is very closely linked to time since immigration, and we were therefore not able to examine effects of time since immigration and country of birth simultaneously.

Since some women had unknown subtype, the subtype-specific incidence rates are somewhat underestimated. However, the proportion with unknown subtype was small (11%) and distributed fairly evenly across regions so we assume that missing subtype was non-informative with respect to immigrant status, and the relative effects unbiased after adjustments. We assessed the impact of unknown subtype by restricting to the diagnosis period 2010–2015 and essentially found estimates to be almost identical to the full study period 2005–2015.

Some immigrants may emigrate back to their country of birth and delay or fail to notify the Norwegian population registry, leading to an overestimation of person years and an underestimation of cancer cases. However, the registration of migration dates at Statistics Norway is generally deemed to be of high accuracy.

If an individual immigrates to Norway with a prevalent cancer, it would be registered as an incident cancer in Norway. However, only 57 immigrants in our cohort had a diagnosis of incident cancer within six months of immigration. We did censor at first emigration to avoid misclassification of individuals who got a diagnosis abroad and returned to Norway.

## Conclusions

In conclusion, we found that immigrants from Eastern Europe, Sub-Saharan Africa and Asia had lower incidence of the luminal A-like subtype, and immigrants from Asia had higher rates of HER2+ tumors, compared to non-immigrants. The rates of TNBC tended to be similar regardless of region of birth, except in women from South-East Asia who had half the rate. Our findings demonstrate that it is important to include immigrant status or country of birth in ascertaining cancer outcomes. Differences in incidence rates of subtypes between immigrant groups are likely multifactorial, including genetic and social factors, lifestyle and health care seeking practices. These differences in tumor characteristics between immigrant groups should be taken into consideration when planning preventive or screening strategies.

## Supplementary Information

**Additional file 1**. Supplementary Tables S1–S3.

## Data Availability

The data that support the findings of this study are available from Statistics Norway and the Cancer Registry of Norway, but restrictions apply to the availability of these data, which were used under license for the current study, and so are not publicly available. Data are however available from the authors upon reasonable request and with permission of Statistics Norway and the Cancer Registry of Norway.

## Abbreviations

ASR: Age-standardized incidence rate
BMI: Body mass index
BRCA: Breast cancer susceptibility gene
CI: Confidence interval
ER: Estrogen receptor
HER2: Human epidermal growth factor 2
ICD: International Classification of Diseases
IHC: Immunohistochemistry
IRR: Incidence rate ratio
PIN: Personal identification number
PR: Progesterone receptor
SES: Socioeconomic status
TNBC: Triple-negative breast cancer
