# Supplementary material for: Incidence of breast cancer subtypes in immigrant and non-immigrant women in Norway
Source: Breast Cancer Res. 2022 Jan 10;24:4. doi: 10.1186/s13058-021-01498-5 (PMC8751256; doi:10.1186/s13058-021-01498-5)
Supplement: Supplementary file 1 — Additional file 1. Supplementary Tables S1–S3. [file 13058_2021_1498_MOESM1_ESM.docx]

**Supplementary Table 1.** Countries of birth included in regions.

| **Region** | **Country of birth** |
| --- | --- |
| High-income countries | Denmark, Finland, Iceland, Sweden, Belgium, France, Ireland, Italy, Netherlands, Portugal, Spain, Great Britain, Switzerland, Germany, Austria, Lichtenstein, Luxembourg, Canada, USA, Australia, New Zealand |
| Eastern Europe | Albania, Bulgaria, Estonia, Greece, Belarus, Croatia, Latvia, Serbia and Montenegro, Poland, Romania, Soviet Union, Lithuania, Moldova, Russia, Czechoslovakia, Turkey, Slovenia, Ukraine, DDR, Hungary, Bosnia-Hercegovina, Makedonia, Slovakia, Czech Republic, Serbia, Montenegro, Kosovo, Cyprus |
| Middle East | Algeria, Egypt, Morocco, Sudan, Tunisia, Iraq, Iran, Lebanon, Palestine, Syria |
| Sub-Saharan Africa | Botswana, Burundi, Comoros, Benin, Equatorial-Guinea, Ivory Coast, Eritrea, Ethiopia, Gabon, Gambia, Ghana, Guinea, Guinea-Bissau, Cameroon, Cape Verde, Kenya, Congo-Brazzaville, Congo, Liberia, Madagascar, Malawi, Mali, Mauritius, Namibia, Niger, Nigeria, Mozambique, Zimbabwe, Rwanda, Senegal, Seychelles, Sierra Leone, Somalia, South Africa, Tanzania, Chad, Togo, Uganda, Zambia, Burkina Faso |
| South Asia | Afghanistan, Bangladesh, Sri Lanka, India, Nepal, Pakistan |
| South-East Asia | Myanmar, Philippines, Indonesia, Thailand, Vietnam |

Eastern Europe includes: Eastern Europe, Baltics and Balkan

Middle East includes: Middle East and North Africa

**Supplementary Table 2.** Incidence Rate Ratios (IRRs) for country of birth and invasive breast cancer subtypes, restricted to the period 2010-2015.

|  | **Age 20-75** | |
| --- | --- | --- |
|  | **Cases/PYR** | **IRR (95% CI)** |
| **All subtypes** |  |  |
| Non-immigrants | 12376/8023506 | 1.00 (ref) |
| High-income | 423/287407 | 1.14 (1.03-1.25) |
| Eastern Europe | 293/416867 | 0.84 (0.75-0.94) |
| Middle East | 96/111018 | 0.97 (0.79-1.19) |
| Sub-Saharan Africa | 48/104411 | 0.67 (0.51-0.89) |
| South Asia | 85/109529 | 0.74 (0.60-0.91) |
| South-East Asia | 135/183734 | 0.78 (0.66-0.93) |
| **Luminal A-like** |  |  |
| Non-immigrants | 7178/8023506 | 1.00 (ref) |
| High-income | 243/287407 | 1.14 (1.00-1.29) |
| Eastern Europe | 156/416867 | 0.81 (0.69-0,95) |
| Middle East | 57/111018 | 1.04 (0.80-1.35) |
| Sub-Saharan Africa | 16/104411 | 0.42 (0.26-0.68) |
| South Asia | 41/109529 | 0.64 (0.47-0.87) |
| South-East Asia | 65/183734 | 0.68 (0.53-0.87) |

Incidence rate ratio (IRR) from Poisson regression. Adjusted for age (5y).

Abbreviations: PYR; person-years, IRR; incidence rate ratio, CI; confidence interval, ref; reference, High-income; Western Europe, USA, Canada, Australia and New Zealand, Eastern Europe; Eastern Europe, Baltics and Balkan, Middle East; Middle East and North Africa.

**Supplementary Table 3.** Incidence Rate Ratios (IRRs) for country of birth and invasive breast cancer subtypes, restricted to women aged 20-40 years.

|  | **Age 20-40** | |
| --- | --- | --- |
|  | **Cases/PYR** | **IRR (95% CI)** |
| **All subtypes** |  |  |
| Non-immigrants | 1155/5550579 | 1.00 (ref) |
| High-income | 47/213110 | 1.02 (0.76-1.36) |
| Eastern Europe | 68/376516 | 0.89 (0.69-1.13) |
| Middle East | 21/109429 | 0.91 (0.59-1.40) |
| Sub-Saharan Africa | 27/108767 | 1.21 (0.83-0.1.78) |
| South Asia | 27/101663 | 1.21 (0.83-1.78) |
| South-East Asia | 37/170044 | 0.94 (0.68-1.30) |
| **Luminal A-like** |  |  |
| Non-immigrants | 430/5550579 | 1.00 (ref) |
| High-income | 22/213110 | 1.29 (0.84-1.98) |
| Eastern Europe | 27/376516 | 0.97 (0.66-1.43) |
| Middle East | 7/109429 | 0.82 (0.39-1.73) |
| Sub-Saharan Africa | 5/108767 | 0.61 (0.25-1.48) |
| South Asia | 10/101663 | 1.22 (0.65-2.29) |
| South-East Asia | 5/170044 | 0.34 (0.14-0.83) |

Incidence rate ratio (IRR) from Poisson regression. Adjusted for age (5y).

Abbreviations: PYR; person-years, IRR; incidence rate ratio, CI; confidence interval, ref; reference, High-income; Western Europe, USA, Canada, Australia and New Zealand, Eastern Europe; Eastern Europe, Baltics and Balkan, Middle East; Middle East and North Africa.
